# Craniopharyngeal duct: a cause of recurrent meningitis

**DOI:** 10.1259/bjrcr.20150022

**Published:** 2015-07-17

**Authors:** S Biswas, C P Millward, A Riordan, A Sinha, S Avula

**Affiliations:** ^1^ Department of Neuroradiology, The Walton Centre for Neurology and Neurosurgery, Liverpool, UK; ^2^ Department of Neurosurgery, Alder Hey Children's Hospital NHS Foundation Trust, Liverpool, UK; ^3^ Department of Paediatric Infectious Disease and Immunology, Alder Hey Children's NHS Foundation Trust, Liverpool, UK; ^4^ Department of Radiology, Alder Hey Children's NHS Foundation Trust, Liverpool, UK

## Abstract

Identification of the cause of recurrent meningitis may pose a diagnostic challenge. Evaluation of a patient with recurrent meningitis calls for meticulous review of skull base structures by cross sectional imaging to exclude any underlying anatomical abnormality. Our case highlights the importance of excluding persistent craniopharyngeal duct, a rare but treatable cause of recurrent meningitis. The isolation of *Streptococcus pneumoniae* in recurrent meningitis may be a clue to the presence of a skull base abnormality. Craniopharyngeal canals have been classified depending on their qualitative and quantitative imaging features. Such imaging based classification is important for identification of patients with associated potential pituitary involvement and also for appropriate surgical planning. Controversy exists as to the approach to surgical treatment of craniopahryngeal duct. The persistent craniopahryngeal duct in our patient was successfully treated by an endoscopic transsphenoidal approach.

## Introduction

Cranial anatomical defects may predispose to recurrent meningitis.^1^ Although uncommon, a persistent craniopharyngeal duct may serve as a conduit for infective organisms to transcend intracranially. We describe a patient who presented with recurrent meningitis and was subsequently investigated to identify any underlying cause.

## Clinical presentation

A 5-year-old girl presented to the emergency department with headache, vomiting, increasing drowsiness and fever. A clinical diagnosis of meningitis was made. She was treated with intravenous antibiotics (ceftriaxone, 1300 mg, once daily) for a period of 14 days and made a full recovery from this episode. A diagnosis of pneumococcal meningitis was made following the identification of *Streptococcus pneumoniae *serotype 23B from the blood and cerebrospinal fluid (CSF) cultures.

15 months later, the patient presented to the emergency department of our hospital again with symptoms suggestive of meningitis. A CT scan of the head was performed to exclude a suspected intracranial abscess and was reported as normal. The patient was treated for meningitis with intravenous antibiotics and made a full recovery. CSF showed an increased white cell count [7160 × 10^6^ l^−1^ (50% neutrophils)]. Blood culture grew *S. pneumoniae* serotype 21 and polymerase chain reaction analysis of CSF was positive for *S. pneumoniae*.

The patient did not have history of concurrent infective illnesses, including middle ear or mastoid infections on either admission. There was no history of other recurrent infections, head injury, CSF rhinorrhoea or otorrhoea. The patient’s perinatal history was uneventful and she had normal developmental milestones. On examination, there were no stigmata of spinal dysraphism.

Following treatment for the second episode of pneumococcal meningitis, the patient was reviewed by the infectious disease team and was commenced on long-term prophylactic antibiotics (amoxicillin) pending further investigations to ascertain an explanation for her recurrent episodes of pneumococcal meningitis.

## Investigation/imaging findings

To investigate the cause of her recurrent meningitis, further immunological tests were performed. The immunoglobulin levels, complement activity, and T and B lymphocyte counts were normal. She continued to have protective levels against the 13 pneumococcal serotypes that she had been vaccinated against (Prevenar 13), although these levels had decreased since they were last measured following her booster dose.

**Figure 1.**
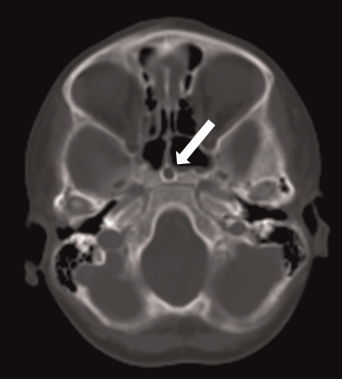
Axial CT scan of the head in bone window showing a round corticated defect (arrow) in the sphenoid body.

**Figure 2.**
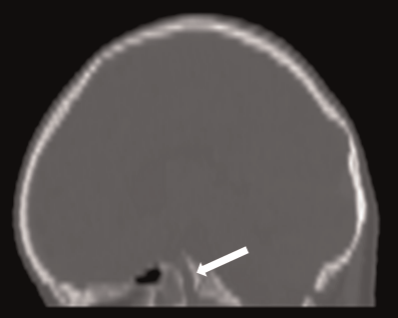
CT scan of the head with sagittal reformat on bone window through the midline shows defect (arrow) in the sphenoid body.

**Figure 3.**
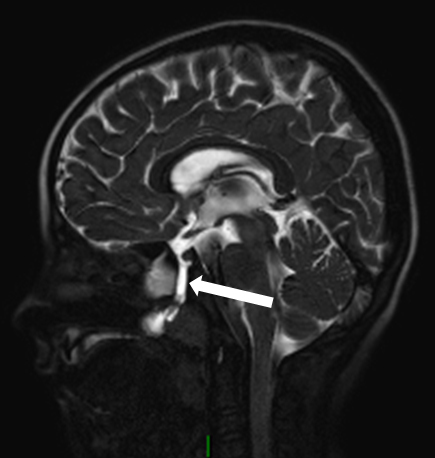
Preoperative MRI. *T*_2_ weighted sagittal image through the midline shows cerebrospinal fluid-filled tract (arrow) communicating between the floor of the sella and the nasopharynx.

The recurrent episodes of meningitis caused by pneumococci were deemed unusual by the infectious diseases team. This prompted the need for further review of radiological investigations to identify a congenital anomaly that would allow *S. pneumoniae* (a commensal in the nasopharynx) access to the CSF. The review of the initial CT scan of the head confirmed the presence of a bony canal, measuring about 6 mm in diameter, extending from the sella, through the sphenoid and opening into the nasopharynx. A diagnosis of a persistent craniopharyngeal duct was made (Figures 1 and 2). An MRI of the pituitary gland demonstrated the defect as a tract from the floor of the sella turcica to the nasopharynx, which was hyperintense on *T*_2_ weighted and hypointense on *T*_1_ weighted sequences. The abnormality was best demonstrated on a heavily *T*_2_ weighted sequence acquired volumetrically, which was performed as a part of a preoperative MRI for surgical planning (Figure 3). There was no evidence of associated tumour or ectopic/herniating pituitary tissue.

## Treatment

The patient underwent an endoscopic transphenoidal surgical repair of the persistent craniopharyngeal duct. This was a joint procedure involving a neurosurgeon and an ear, nose and throat surgeon. Endoscopic approach through both nostrils to gain bimanual access to the tract was performed. Intraoperative image guidance using CT and MRI was used to accurately identify the craniopharyngeal tract. The sphenoid sinus was exposed and the floor of the sella turcica was identified. The floor of the pituitary fossa was drilled to expose the basal dura, which was traced to the craniopharyngeal tract. This was isolated and divided. Fat graft (harvested from the abdomen) and fibrin glue were used to repair the defect in the pituitary fossa. The distal end of the craniopharyngeal tract was seen ending in a blind sac in the nasopharynx. This was punctured and obliterated with electrocautery.

## Outcome and follow-up

**Figure 4.**
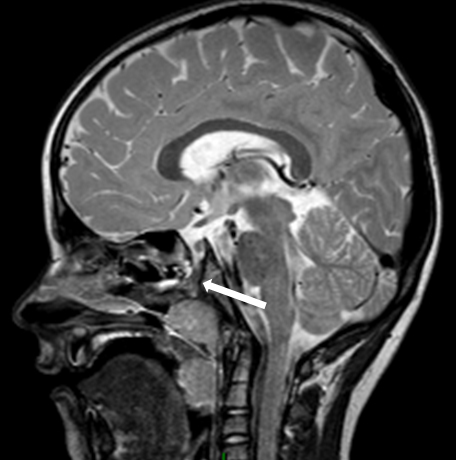
Postoperative MRI. *T*_2_ weighted sagittal image shows obliteration of the tract by soft-tissue material (arrow).

A postoperative MRI (Figure 4) showed obliteration of the craniopharyngeal duct. Our patient has made a full recovery, stopped antibiotic prophylaxis and has not had any further episodes of meningitis.

## Discussion

Several conditions may predispose to the development of recurrent meningitis. These include anatomical abnormalities, immunodeficiency disorders and chronic parameningeal infections.^1^ Anatomical defects may be acquired or congenital. Acquired causes may be as a result of head injury causing CSF fistulae or secondary to neoplasms causing erosion of the skull base. Congenital abnormalities may include a wide spectrum of defects in the skull base. These defects may range from craniopharyngeal duct to large cephalocoeles with craniofacial defects.^2^

Craniopharyngeal duct is a bony channel that connects the floor of the sella turcica, along the midline, to the nasopharynx.^3^ It can persist as a corticated defect of the midline sphenoid body. Craniopharyngeal duct is believed to arise owing to a defect in the normal development of the pituitary. The adenohypophyseal (Rathke’s) pouch is formed at about the fourth week of gestation from the primitive mouth cavity (stomatodeum) and extends towards the brain.^2,4^ Around the fifth to sixth week, the pouch elongates further, forming an elongated stalk between the pouch and the stomatodeum The cartilaginous skull base forms at around the sixth to seventh week of gestation, obliterating the adenohypophyseal stalk.^4^ Incomplete fusion of the cartilaginous elements results in incomplete obliteration of the adenohypohyseal stalk, which persists as the craniopharyngeal duct or canal, extending from the sella turcica to the nasopharynx.^2,4,5^ Some authors have proposed that the craniopharyngeal duct represents a vascular channel.^3^ However, the histological demonstration of normal as well as adenomatous pituitary tissue in craniopharyngeal ducts argue strongly in favour of the craniopahryngeal duct being of pituitary origin.^4,5^

Small craniopharyngeal canals have been reported to occur in up to 0.42% of the asymptomatic population.^6^ Currarino et al^7^ classified the abnormality on the basis of size: the hypophyseal channel or the small craniopharyngeal canal, which have a maximum width of 15 mm, and the large craniopharyngeal canal or transspehnoidal channel, which are more commonly associated with other craniofacial abnormalities such as encephalocoeles, cleft palate and lips and such. More recently, a classification of the craniopharyngeal duct has been proposed by Abele et al^4^ based on the size and spectrum of other associated pathologies (e.g. pituitary adenomas, craniophahryngiomas, teratomas and gliomas). In the case series forming the basis of the classification system, the quantitative evaluation showed a significant difference (*p *< 0.0001) in the anteroposterior diameters of types 1, 2 and 3 canals, thus described as small, medium and large canals, respectively. The classification is summarized in Table 1. On the basis of this classification system, the craniopharyngeal duct described in our case can be categorized as a type 3A owing to its size (6 mm), orthoptic position of the pituitary and the absence of any associated tumour. Such an imaging-based classification can identify patients with potential pituitary dysfunction. This classification system categorically takes into account the presence or absence of associated tumours, which is important in surgical planning. The classification/characterization of the ducts also plays a crucial role in preventing iatrogenic complications, such as post-surgical hypopituitarism or CSF leak.

**Table 1.** Classification of craniopharyngeal ducts

Type	Features	Antero-posterior diameter
Type 1	Incidental canals	Range: 0.7–1.1 mm Median: 0.8
Type 2	Canals with ectopic adenohypophysis	Range: 3.5–4.4 mm Median: 3.9
Type 3A	Canals with cephaloceles	Range: 5.9–31.0 mm Median: 9.0
Type 3B	Associated tumours (*e*.*g*. pituitary adenoma, craniopharyngioma, dermoid, teratoma and glioma)	As type 3A
Type 3C	Features of both type 3A and B	As type 3A

Brain imaging is indicated only when the diagnosis of meningitis is clinically uncertain and when there is unusual presentation or suspected complication (reduced level of consciousness or focal neurological signs).^8^ In cases of meningitis with predisposing factors, such as skull base fracture, middle ear and mastoid infection, imaging is necessary to identify the underlying cause. Our case illustrates the importance of reviewing the skull base in patients with recurrent meningitis, as early identification and treatment of an abnormal communication can potentially prevent further intracranial infections.

Standard CT imaging of the head as well as MR sequences may at times be suboptimal for demonstrating a narrow craniopharyngeal duct. A high-resolution CT scan of the skull base postprocessed with bone algorithm and viewed with wide (bone) window may be necessary to demonstrate a narrow craniopharyngeal duct. MRI is necessary to localize the pituitary and exclude the presence of associated neoplasms. A sagittal heavily *T*_2_ weighted sequence acquired volumetrically may also be helpful in further characterizing the abnormality, as in our case.

Correlation of the type of organism isolated from the CSF and/or blood in recurrent meningitis and the site of anatomical defect has been described.^9^ There is a predisposition of *S. pneumoniae* in patients with intracranial encephalocoeles. The isolation of *S. pneumoniae* from the CSF and blood on two occasions in our patient prompted the review of the CT scan, which led to the diagnosis of the persistent craniopharyngeal duct.

There are different approaches to the repair of a craniopharyngeal duct that include transcranial, transoral–transpalatal and transsphenoidal endoscopic, with some controversy existing regarding the choice of approach.^5^ Our patient was treated with transsphenoidal endoscopic approach with good outcome.

## Learning points

1. The skull base is a “review area” in imaging of patient with intracranial infection, particularly in cases of recurrent meningitis, in order to exclude underlying defects such as a persistent craniopharyngeal duct.
2. In recurrent meningitis, the isolation of *S. pneumoniae *may point towards the presence of such a defect.
3. High-resolution CT scan of the head with reconstruction in bone algorithm can identify a persistent craniopahryngeal duct, and an MRI is necessary to gather important information such as position of the pituitary and presence/absence of associated neoplasms.
4. A useful classification system of craniopahryngeal canals based on qualitative and quantitative criteria has been described.
5. Although controversy exists regarding the surgical approach, our case describes successful treatment using an endoscopic transsphenoidal approach.
